# Broadly reactive aptamers targeting bacteria belonging to different genera using a sequential toggle cell-SELEX

**DOI:** 10.1038/srep43641

**Published:** 2017-03-08

**Authors:** Min Young Song, Dung Nguyen, Seok Won Hong, Byoung Chan Kim

**Affiliations:** 1 Center for Environment, Health and Welfare Research, Korea Institute of Science and Technology (KIST), Seoul 02792, Republic of Korea; 2 Department of Energy and Environmental Engineering, University of Science and Technology (UST), Hwarangno 14-gil 5, Seongbuk-gu, Seoul 02792, Republic of Korea; 3 Center for Water Resources Cycle Research, Korea Institute of Science and Technology (KIST), Hwarangno 14-gil 5, Seongbuk-gu, Seoul 02792, Republic of Korea

## Abstract

Conventional cell-SELEX aims to isolate aptamers to a single unique target bacteria species. We propose a method to isolate single-stranded DNA aptamers that have broad reactivity to multiple bacterial targets belonging to different genera. The key of the proposed method is that targets of interest are changed sequentially at each SELEX round. The general scheme was examined using six bacteria from different genera, *Escherichia coli, Enterobacter aerogenes, Klebsiella pneumoniae, Citrobacter freundii, Bacillus subtilis*, and *Staphylococcus epidermidis* (four gram-negative and two gram-positive bacteria). In the first round of SELEX, the DNA library was incubated with *E. coli* and amplicons bound to *E. coli* were separated. The amplicons were sequentially separated by incubation with *E. aerogenes, K. pneumoniae, C. freundii, B. subtilis*, and *S. epidermidis* at each SELEX. The amplicons obtained using the last bacterial species were incubated again with the first bacterial species and this loop was repeated two more times. We refer to this method as sequential toggle cell-SELEX (STC-SELEX). The isolated aptamers had dissociation constants of 9.22–38.5 nM and had no affinity to other bacteria that were not included in STC-SELEX. These results demonstrate the potential to isolate aptamers with broad affinity to bacterial taxa in different genera.

Aptamers represent an important technical advance by enabling new diagnostic and therapeutic strategies based on high-affinity binding to targets^1^,^2^,^3^,^4^,^5^. Systematic evolution of ligands by exponential enrichment (SELEX) is used for aptamer screening and characterization to find receptors that have very high specific affinities to targets of interest, such as small molecules, proteins, or cells since its invention around 1990^4^,^6^,^7^,^8^. Studies of aptamer-based receptors have focused on finding highly functional nucleic acids that recognize a single unique target, minimizing cross-reactivity to unwanted targets that share epitopes with structural similarity^9^. For example, aptamers are capable of distinguishing glucose, fructose, and galactose, which share similar molecular structures^10^. Excessive specificity, however, is not always desired when cross-reactivity or broad reactivity is needed to detect multiple targets with similar or different structures^11^. For example, broadly reactive receptors, such as antibodies, are still necessary to cure viral infections^12^,^13^.

Aptamers are often compared to antibodies. Before the development of protocols to generate aptamers, antibodies made profound contributions to a wide range of fields owing to their diagnostic and therapeutic applications^14^. For many applications, antibodies need to be very specific to target antigens. However, cross-reactivity or broad reactivity is desired to cure or diagnose diseases. For example, broadly neutralizing antibodies can bind to and neutralize multiple, rapidly mutating viral strains, like influenza virus, HIV, or HCV, to reduce infection, irrespective of genotype^12^,^15^,^16^. Broadly neutralizing antibodies have some limitations with respect to obtaining target specimens and appropriate cellular models, production time, confirmation of reproducibility, and storage stability. Despite production difficulties, the screening and isolation of broadly neutralizing antibodies are of vital importance for the treatment of viral-induced diseases. Like antibodies, aptamers can be used to isolate cross-reactive or broadly reactive receptors for targets that share similar epitopes. Recently, aptamers that have broad binding affinities to various targets have been isolated for human thrombin and porcine thrombin^11^, the family of zinc finger proteins^17^, multiple type strains of *Streptococcus pyogenes*^18^, and subtypes of hemagglutinin proteins of the influenza A virus^19^. Although these examples show the potential to isolate aptamers that have broad reactivity, the targets in previous studies shared similar epitopes or had high structural similarity. Therefore, it is unclear whether aptamers can be isolated for targets spanning distinct evolutionary taxa, such as bacteria belonging to different genera.

Using current cell-SELEX methods, aptamers with high affinity and specificity to only a single target bacterial species are isolated^20^,^21^. Specifically, the cell-SELEX method includes the following steps: (1) binding between cells of a single target bacterial species and a nucleic acid library consisting of random sequences; (2) removing unbound sequences and obtaining nucleic acids capable of binding to the target; and (3) amplifying the nucleic acids that bound to the target for subsequent rounds of selection. These steps are repeated several times to discover nucleic acid aptamers with high affinity and specificity for target bacterial species^20^,^22^. Most cell-SELEX methods are applied to only one target, despite the coexistence of various microorganisms in many environments, clinical settings, or laboratories. It is sometimes useful to detect groups of bacteria simultaneously, rather than individually. For example, the most common bacteria causing beer spoilage during the brewing process are *Lactobacillus, Pediococcus*, and *Acetobacter*^23^. To ensure a high-quality product, control measures are necessary to prevent the contamination of those bacterial groups during the brewing process. Accordingly, broadly reactive receptors that can simultaneously identify bacterial contaminants in multiple genera are very useful. However, methods to identify DNA aptamers that have broad affinity to various microorganisms have not been developed.

Here, we describe the *in vitro* isolation of aptamers that have broad affinity against bacteria in different genera. For this, we suggested sequential toggle cell-SELEX (STC-SELEX) in this study. The key development in the proposed STC-SELEX method is that different targets of interest are used in each round of aptamer selection. The isolated aptamers were characterized by their affinity and broad reactivity. Aptamers obtained using this approach can be expected to share the sequences that can bind with all bacteria that are participated in the STC-SELEX process, however, exact targets on the surface of bacteria should be studied further in terms of interaction with aptamers.

## Results and Discussion

### Sequential Toggle Cell (STC)-SELEX and Sequences of Aptamers

**Figure 1:**
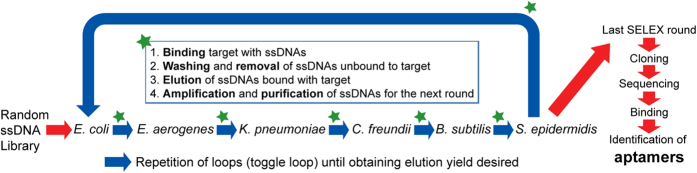
STC-SELEX process. The STC-SELEX process starts with a random DNA library. After incubation of the random library with the first target bacteria, the isolation step is performed to obtain DNA that bound to the target. After washing to remove unbound DNA, elution of the bound DNA with the target, amplification and purification, the process is repeated using the next target bacteria. After repetition of this process using all targets, the selection process is repeated, beginning with the first target. This SELEX loop is repeated until the desired elution yield is obtained.

**Figure 2:**
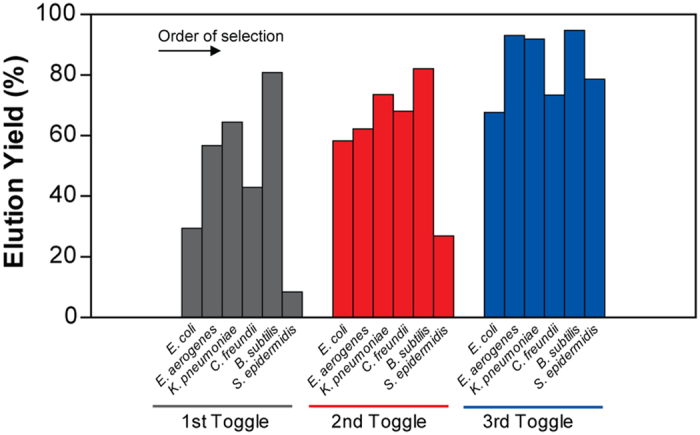
Elution yield of ssDNA at each SELEX step during STC-SELEX.

**Figure 3:**
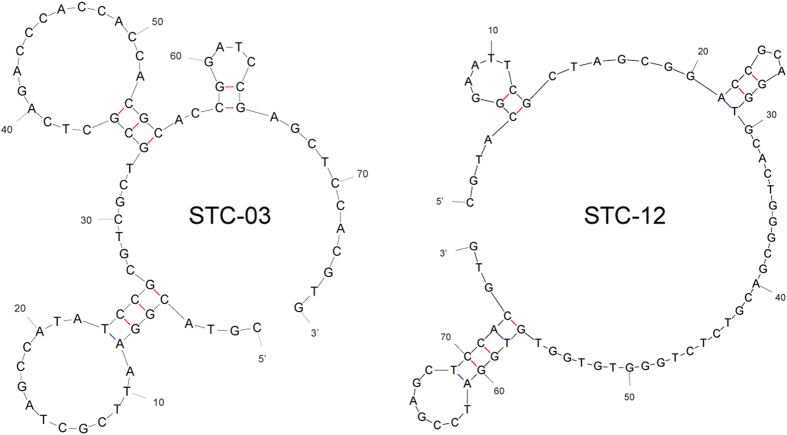
2D structure of two isolated aptamers (STC-03 and STC-12) that had broad reactivity to six bacteria included in STC-SELEX.

Figure 1 illustrates the procedure for screening single-stranded DNA (ssDNA) aptamers that have broad affinity to six bacteria in different genera (*Escherichia coli, Enterobacter aerogenes, Klebsiella pneumoniae, Citrobacter freundii, Bacillus subtilis*, and *Staphylococcus epidermidis*). The SELEX process was started using a library containing random ssDNA fragments for the isolation of aptamers. In the first round of *in vitro* selection, *E. coli* cells were incubated with the starting library. Unbound DNA was removed and bound ssDNA was recovered and amplified to obtain enriched ssDNAs by PCR for the next round of selection. In the second round of selection, the enriched ssDNAs that bound to *E. coli* were incubated with another target, *E. aerogenes*. This process was repeated using *E. aerogenes, K. pneumoniae, C. freundii, B. subtilis*, and *S. epidermidis*. We repeated this cycle, using targets from *E. coli* to *S. epidermidis*, three times (3 toggle loops) until the desired ssDNA elution yield was obtained. We called this SELEX process Sequential Toggle Cell-SELEX (STC-SELEX) (Fig. 1). We carried out 18 rounds of SELEX and obtained an elution yield of ~80% in the final SELEX round. In the first toggle loop, the elution yield of ssDNAs increased from *E. coli* to *K. pneumoniae* to over 60%. The elution yield decreased to 40% in the selection process using *C. freundii* and then increased to 80% using *B. subtilis*. However, the elution yield decreased abruptly to less than 10% after selection with *S. epidermidis*. These results indicate that most ssDNAs with broad affinity to *E. coli, E. aerogenes, K. pneumoniae, C. freundii*, and *B. subtilis* were rejected at the stage with *S. epidermidis*. The increasing and decreasing patterns of elution yield in each toggle round were similar. The overall elution yields increased gradually during toggle loops. After the second toggle round the elution yield was ~30% and the elution yield was ~80% at the final SELEX process (Fig. 2), indicating that the repeated looping process can help obtain highly reactive aptamers to all target bacteria included in the process. The STC-SELEX strategy can theoretically be applied to unlimited targets including closely or distantly related bacterial strains via iteration *in vitro* and this strategy can be generalized to other combinatorial target groups to isolate broadly reactive aptamers. The products were amplified by PCR after the 18th round of selection (3 loops using 6 different bacteria) and cloned, resulting in 42 total clones. After sequencing analysis, 15 different ssDNA clones, excluding overlapping sequences, were obtained. Table 1 shows a summary of the 15 ssDNA sequences after STC-SELEX. We selected two sequences, STC-03 and STC-12, for an additional affinity analysis because they had a high number of isolated sequences (STC-03, 10 out of 42 clones; STC-12, 8 out of 42 clones). STC-01, STC-02, STC-04, and STC-05 shared a conserved region with STC-03: ATA TCC GYG TCG CTG CGC TCA GAC CCA CC (where Y indicates a C or T). STC-10 and STC-11 also shared a conserved region with STC-12: GAC CGC AGG TGC ACT GGG CGA CGT CTC TGG GTG TGG TG. Therefore, we inferred that these two conserved sequences are essential for conferring the broad affinity to six bacterial taxa simultaneously. The secondary structures of the two sequences STC-03 and STC-12 were predicted using Mfold^24^. The two aptamers showed three bulb-like stem-loop structures (Fig. 3).

### Binding Affinity of Aptamers

**Figure 4:**
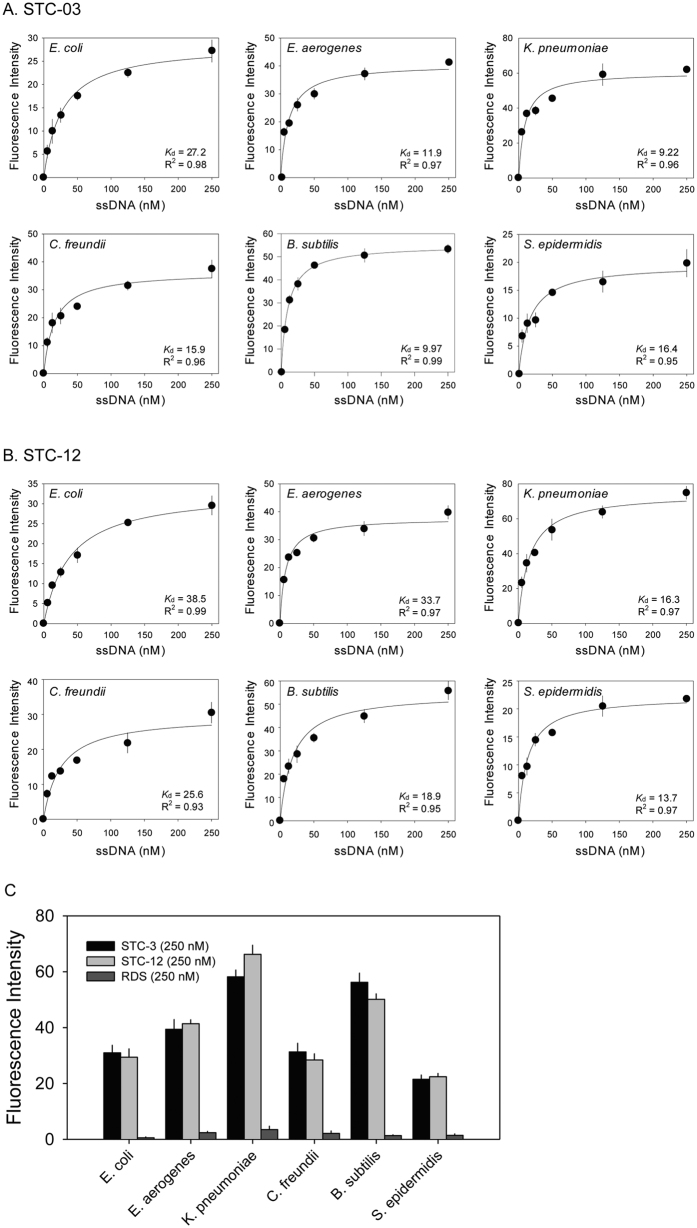
Binding saturation curve of the aptamers (**A**) STC-03 and (**B**) STC-12 to each target, and (**C**) comparison of binding intensity of STC-03, STC-12, and random DNA (250 nM) to each target. Nonlinear regression curves were fit to the intensity data of (**A**) and (**B**).

**Figure 5:**
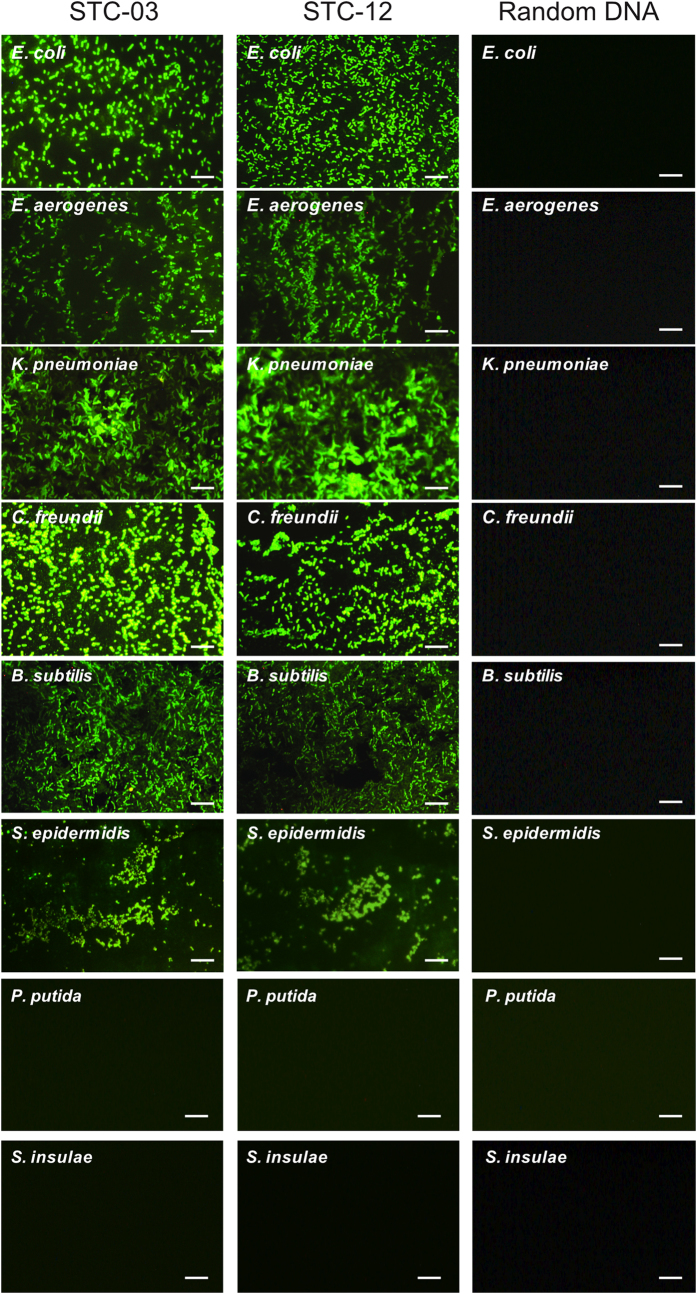
Fluorescence microscopy images of bacteria after mixing and washing with fluorescent-tagged aptamers, STC-03 (left column), STC-12 (middle column) or random DNA (right column). White scale bars denote 10 μm.

We next investigated the binding affinities of the two sequences (STC-03 and STC-12) to 6 different bacteria. Fluorescently labelled aptamer sequences were tested against the 6 bacteria included in the STC-SELEX procedure. Various concentrations of each sequence labelled with FAM dye were incubated with 6 different bacteria using equal cell numbers. Both selected aptamer sequences showed typical binding saturation curves from a fluorescence analysis, suggesting preferential binding of the aptamer sequences to all 6 taxa (Fig. 4(A) and (B)). Curves were fitted using non-linear regression based on the following equation: F = *B*_max_ × C/(*K*_d_ + C) (where F indicates the fluorescence intensity, *B*_max_ indicates the maximum intensity of binding, *K*_d_ indicates the dissociation constant, and C indicates the ssDNA aptamer concentration). Dissociation constants (*K*_d_) ranging from 9.22 nM to 38.5 nM were observed for each bacterium. Although the dissociation constants varied, saturation binding curves for the two sequences in all assays were observed. STC-03 showed better affinity to *E. aerogenes, K. pneumoniae, C. freundii*, and *B. subtilis*, while STC-12 showed better affinity to *E. coli* and *S. epidermidis*. It was confirmed that both STC-03 and STC-12 had high affinity to all 6 bacteria. These dissociation constants indicated favourable binding affinities to all 6 bacteria compared to other aptamers isolated from unique target^20^,^25^. The negative control test with random ssDNA (250 nM) didn’t show significant fluorescence intensities compared to both STC-03 and STC-12 (Fig. 4(C)). In addition, directly observed the binding of the STC-03 and STC-12 aptamers labelled with fluorescent dye to each bacterium using fluorescent microscopy. Based on the estimated affinity values, both aptamers were expected to induce fluorescence reflecting the morphology of the bacteria. As shown in Fig. 5, fluorescent dye-labelled STC-03 and STC-12 showed bright fluorescence and clear cell morphologies to the six target bacteria while didn’t show fluorescence to the two non-target bacteria (*P. putida* and *S. insulae*). The negative control tests (six target bacteria or two non-target bacteria incubated with fluorescent dye-labelled random ssDNA) didn’t give any significant fluorescence signals. Fluorescent *in-situ* hybridization (FISH) usually uses oligonucleotide probes that complement the small subunit rRNA sequence and have fluorescent labels to identify specific bacteria^26^. FISH techniques, however, require a cell penetration strategy for the labelled oligonucleotide probes. Considering the results presented in Fig. 5, we suspected that the aptamers isolated using STC-SELEX would be effective probes to identify bacteria of interest by recognizing cellular surface molecules, without requiring penetration of bacterial cells.

**Figure 6:**
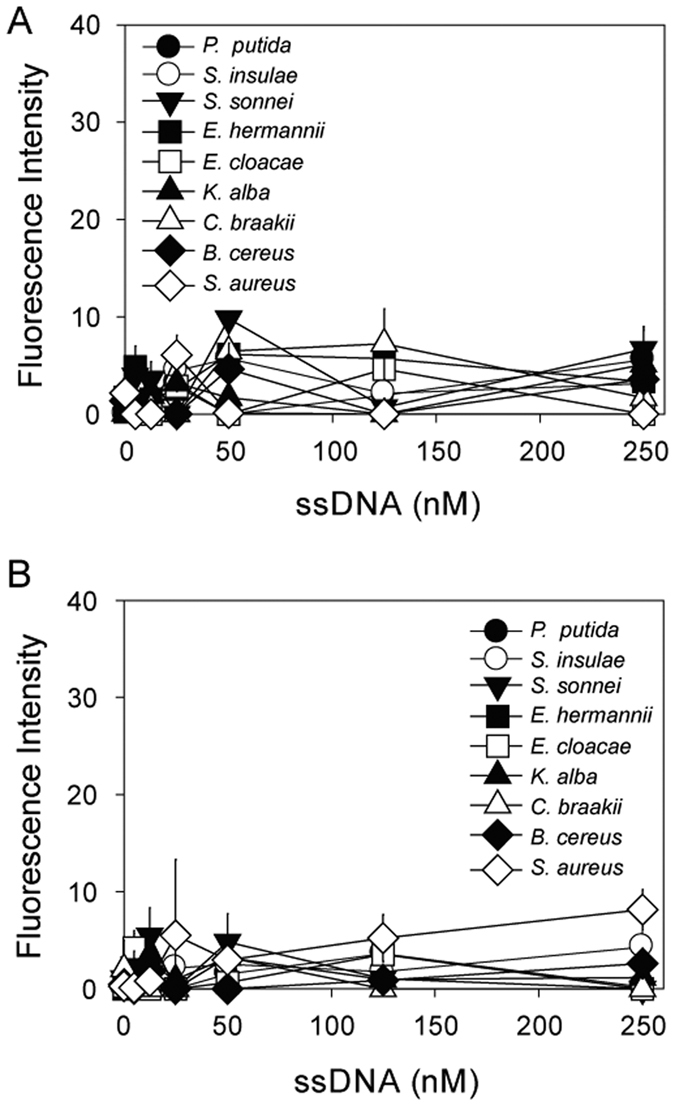
Binding results for STC-03 (**A**) and STC-012 (**B**) with respect to *P. putida, S. insulae, S. sonnei, E. hermannii, E. cloacae, K. alba, C. braakii, B. cereus*, and *S. aureus* which were not involved in the STC-SELEX process.

For the two ssDNA aptamers, a selectivity analysis was performed using nine microorganisms that were not included in the STC-SELEX procedure, i.e., *P. putida, S. insulae*, and *S. sonnei* that were not under same genus of six bacteria included during STC-SELEX process in this study or *E. hermannii, E. cloacae, K. alba, C. braakii, B. cereus*, and *S. aureus* that were under same genus (but not same species) of six bacteria. Similar to the affinity test with 6 different bacteria, we tested the binding affinity of these nine bacteria with either the STC-03 or STC-12 aptamer. As shown in Fig. 6, the fluorescence intensities for STC-03 and STC-12 binding on the nine bacteria were very low and a saturation curve could not be obtained. The dissociation constants for STC-03 or STC-12 to nine bacteria could not be obtained. These results imply that STC-03 and STC-12 have affinity to only the bacteria included in the STC-SELEX procedure.

Tremendous efforts have been made to isolate specific aptamers to unique targets for a range of applications, including disease diagnosis and treatment as well as the elimination of micro-pollutants^4^,^6^,^27^. This is the first report of aptamers that were developed for the simultaneous recognition of multiple bacterial targets belonging to different genera. We developed a selection method to obtain highly specific ssDNA aptamers that are capable of commonly and selectively binding to multiple targets, i.e., two or more bacteria, simultaneously. To identify these aptamers, we sequentially toggled all target bacteria during the SELEX process. This method, which we refer to as STC-SELEX, includes the following key steps: (a) mixing at least one target with a random nucleic acid library, (b) removing unbound nucleic acids, (c) separating nucleic acids that bound to the target; (d) amplifying the nucleic acids via PCR, and (e) repeating steps a–d with the next target bacteria. This strategy is similar to the typical Cell-SELEX process. The main difference that enables the isolation of broadly reactive aptamers is that we changed the target sequentially after each cycle of SELEX (Fig. 1). When one loop of this procedure from the first target to the last target is finished, the SELEX process is repeated, beginning with the first target. Repetition of the toggle flow until around 90% elution yields are observed is sufficient to obtain clones for sequencing to identify aptamer candidates. We performed 18 individual SELEX rounds (3 loops with 6 different bacteria). This procedure can be used to obtain an aptamer that is applicable to the simultaneous detection of two or more bacterial species that are dominant within a limited environment. Accordingly, it is possible to develop broadly reactive aptamers that are capable of generally diagnosing targets in populations and a universal identification system. STC-SELEX can also provide a method for manufacturing aptamers capable of simultaneously detecting or diagnosing various bacteria belonging to the same or different genera. This is particularly useful for pathogen infections in the blood, food, or environment when other diagnostic methods (e.g., PCR, FISH, or cultivation) for each kind of bacteria make the diagnosis complex. The universal identification of bacteria in certain environments has not been practical owing to a lack of appropriate universal receptors. We hope that STC-SELEX is applied to produce customized universal receptors for specific environmental conditions.

### Selectivity of Aptamers

In this study, we used 6 different bacteria (as model targets to verify STC-SELEX), including four gram-negative bacteria (*E. coli, E. aerogenes, K. pneumoniae*, and *C. freundii*) and two gram-positive bacteria (*B. subtilis* and *S. epidermidis*), to isolate broadly reactive aptamers. The two types (gram-positive and gram-negative) of bacteria can be differentiated based on structural differences of the cell walls. Gram-positive bacteria have a peptidoglycan layer, instead of an outer cell membrane, which is found in gram-negative bacteria. Most gram-negative bacteria are pathogenic because lipopolysaccharides on the peptidoglycan layer are highly toxic and antigenic components, while many gram-positive bacteria are non-pathogenic. Owing to their pathogenic properties, there have been efforts to isolate cross-reactive or broadly reactive antibodies against gram-negative bacteria^28^. Some studies have attempted to isolate monoclonal antibodies against multiple gram-positive bacteria^29^. Additionally, the binding of natural antibodies that are produced in the apparent absence of antigenic stimulation to both gram-negative and gram-positive bacteria has been investigated^30^. Some efforts have been made to isolate cross-reactive aptamers against human and pig thrombin, or similar bacterial strains. However, there are no reports of the isolation of aptamers or antibodies that show broadly reactive binding to both gram-negative and gram-positive bacteria belonging to different genera. The *in vitro* selection process of SELEX makes it easy to obtain broadly reactive aptamers that can bind to either gram-negative bacteria or gram-positive bacteria. The results of this study suggest that this method is promising for obtaining broadly reactive aptamers against various bacteria simultaneously, but additional *in vitro* studies are needed to establish the validity of the process for aptamer production. Additionally, molecular structural studies to find the binding domains of aptamers on the surfaces of multiple bacteria are needed. Aptamers with broad reactivity among species or genera will greatly facilitate the simultaneous investigation of some bacterial community groups.

In summary, broadly reactive aptamers that can bind to either gram-negative or gram-positive bacteria belonging to different genera were successfully isolated using the STC-SELEX method. In particular, the STC-SELEX method can be used isolate aptamers by toggling targeted bacterial cells during the SELEX process until the desired elution yield is obtained. We applied the method using six different bacteria, including four gram-negative and two gram-positive bacteria, and isolated broadly reactive aptamers that have binding affinity to all six bacteria, with relevant dissociation constants (in the nanomolar range). The STC-SELEX process developed in this study can be modified to isolate aptamers that can bind to other microbial communities. Accordingly, it is a potentially useful method to find broadly reactive aptamers with affinity to various members of microbial communities, rather than to individual targets.

## Methods

### Bacterial Strains and Culture Media

*Escherichia coli (E. coli*, KCTC 2571), *Escherichia hermannii (E. hermannii*, KCTC 22526), *Enterobacter aerogenes (E. aerogenes*, KCTC 2190), *Enterobacter cloacae (E. cloacae*, KCTC 1685), *Klebsiella pneumoniae (K. pneumoniae*, KCTC 2208), *Klebsiella alba (K. alba*, KCTC 12878), *Citrobacter freundii (C. freundii*, KCTC 2006), *Citrobacter braakii (C. braakii*, KCTC 2006), *Bacillus subtilis (B. subtilis*, KCTC 1022), *Bacillus cereus (B. cereus*, KCTC 3674), *Staphylococcus epidermidis (S. epidermidis*, KCTC 1917), *Staphylococcus aureus (S. aureus*, KCTC 1621), *Pseudomonas putida (P. putida*, KCTC 1639), *Sphingomonas insulae (S. insulae*, KCTC 12872) and *Shigella sonnei (S. sonnei*, KCTC 2518) were obtained from the Korean Collection for Type Culture (KCTC, Jeongeup-si, Korea). Nutrient broth and nutrient agar were purchased from Becton & Dickinson, Co. (Franklin Lakes, NJ, USA). *E. coli, S. epidermidis, K. pneumoniae, K. alba S. sonnei, E. hermannii, C. braakii, and S. aureus* were cultivated at 37 °C, *E. aerogenes, E. cloacae, C. freundii, B. subtilis, and B. cereus* were cultivated at 30 °C, and *P. putida and S. insulae* were cultivated at 25 °C. All bacterial cells were grown in nutrient broth medium (5.0 g of peptone and 3.0 g of beef extract in 1 L of distilled water) under aerobic conditions.

### Sequential Toggle Cell (STC)-SELEX Process

The random single-strand DNA (ssDNA) library and primers for PCR were purchased from Genotech (Daejeon, Korea) and dissolved in distilled water to produce a 10 μM solution. The 74-nt oligonucleotide ssDNA library consisted of an N40 randomized region flanked on both sides by an 18-nt primer region for PCR (5′-CGT ACG GAA TTC GCT AGC-N40-GGA TCC GAG CTC CAC GTG-3′ (76-mer)). The following reverse primer modified with biotin was used for the STC-SELEX process (biotin-modified reverse primer: 5′-biotin-CAC GTG GAG CTC GGA TCC-3′ (18-mer)). All bacteria were cultured from a single colony in separate liquid medium overnight. The cells were then subcultured in a second set of tubes (with a 1:100 inoculum-to-media ratio) and were cultured to reach approximately 1 × 10^8^ CFU/mL. The cultured bacteria were recovered by centrifugation (13,000 rpm) at room temperature for 10 min and washed three times with 1× phosphate-buffered saline (PBS, pH 7.0). The washed bacteria were re-suspended in 1 mL of binding buffer (1× PBS, 0.1 mg/mL salmon sperm DNA, 1% bovine serum albumin (BSA), and 0.05% Tween-20).

For the first round of selection, the initial random ssDNA library (183 pmol) was mixed with 10^7^ *E. coli* cells suspended in binding buffer. The mixture was incubated in a Thermomixer (Eppendorf, Hamburg, Germany) with constant shaking (1,000 rpm) at room temperature for 1 h. The ssDNA that did not bind with *E. coli* in the supernatant was discarded by washing with 1 mL of 1× PBS (pH 7.0) three times. The ssDNA bound to *E. coli* was recovered by centrifugation at 13,000 rpm for 10 min at room temperature. To separate the bound ssDNA from bacterial cells, it was re-suspended with 100 μL of autoclaved water (DNase free), heated at 95 °C for 10 min, and cooled immediately to 4 °C for 10 min. The ssDNA in supernatant was recovered by centrifugation (13,000 rpm) at room temperature for 10 min and then the supernatant was filtered using centrifugal filter units (Amicon Ultra-0.5 mL 50 K centrifugal filter Units, Millipore Ireland Ltd., Cork, Ireland) to remove salmon sperm DNA, BSA, or other proteins that might be still existed in ssDNA mixture isolated. The 260/280 absorbance ratio of samples was checked to assess the purity of ssDNA eluted. Elution yield was calculated by comparing the ssDNA concentration before and after binding to target bacterial cells using the ND 1000 Spectrophotometer (NanoDrop, Thermo SCIENTIFIC, Wilmington, DE, USA). The recovered ssDNA was amplified by PCR for enrichment using an unmodified forward primer and a reverse primer modified by biotin at the 5′ region. All PCR products were purified using the Qiagen MinElute PCR Purification Kit (Qiagen Inc., Hilden, Germany). To separate the ssDNAs from the dsDNAs, magnetic beads (MBs) coated with streptavidin (Life Technology) were used. The PCR product (50 μL) and MB (50 μL, 10 mg/mL) mixture was incubated at room temperature for 30 min and then separated and washed three times using a magnet with 1× PBS (pH 7.0). The MBs bound to the PCR products were incubated with 500 μL of NaOH (200 mM) at room temperature for 10 min, and ssDNAs in the supernatant were then separated using a magnet. The recovered ssDNAs were purified and concentrated using centrifugal filter units (Amicon Ultra-0.5 mL 10 K centrifugal filter Units, Millipore Ireland Ltd., Cork, Ireland) for the next round of the STC-SELEX process. For the next round of selection, the ssDNAs isolated from the first round with *E. coli* were mixed with 10^7^ *E. aerogenes* cells as the second target. The procedure for the isolation of the ssDNAs bound to *E. aerogenes* was the same with the first-round selection. Sequentially, ssDNAs were isolated against *K. pneumoniae, C. freundii, B. subtilis*, and *S. epidermidis* following the same procedure. After one loop of isolation of ssDNAs from *E. coli* to *S. epidermidis*, the ssDNAs were incubated with *E. coli* again. This procedure was repeated for three loops (18 isolation procedures). After the last round, the isolated ssDNAs were amplified to obtain dsDNA using the unmodified primer sets. They were cloned using a TOPO TA Cloning Kit (Invitrogen, Carlsbad, CA, USA) for sequencing, and transformed into *E. coli* DH10B cells (Invitrogen). Colonies suspected of having the isolated ssDNAs were chosen randomly, and the plasmid DNA was purified using a Miniprep Kit (Qiagen Inc., Hilden, Germany). The inserted fragments were confirmed using restriction enzymes and PCR amplification. The sequences of the aptamer candidates were determined by Genotech. The secondary structure of each sequence was predicted using Mfold^24^.

### PCR Amplification

PCR was performed using the GeneAmp PCR System 9700 (Applied Biosystems, Foster City, CA, USA). The PCR mixture contained 5 μL of template ssDNA (~15 ng), 2.5 μL of each primer (10 μM), 25 μL of the PCR master mix solution (AmpliTaq Gold Fast PCR Master Mix, Applied Biosystems, Barcelona, Spain), and 15 μL of autoclaved water. The PCR conditions for ssDNA amplification were 95 °C for 5 min for denaturation, followed by 8 cycles of denaturation at 95 °C for 30 s, annealing at 56.3 °C for 30 s, and extension at 72 °C for 10 s, and a final extension of 72 °C for 5 min for the next round of STC-SELEX selection. After PCR, the reaction products were confirmed by gel electrophoresis using 2.0% agarose gel in 1× TAE (Tris-acetate-EDTA) buffer at 100 V for 40 min. The gels were stained with 0.5 mL of GelRed Nucleic Acid (Biotium, Inc., Hayward, CA, USA) in water and observed under UV light.

### Binding and Imaging Analysis for Aptamers

Affinity and specificity were investigated by binding aptamers to six target bacterial cells (*E. coli, E. aerogenes, K. pneumonia, C. freundii, B. subtilis*, and *S. epidermidis*) individually. Each bacterial species (~10^7^ cells suspended in 1× PBS (100 μL)) was incubated with various concentrations (0, 5, 12.5, 25, 50, 125, and 250 nM in final binding reaction) of 3′-FAM dye-labelled aptamers (in 1× PBS (100 μL)) in a Thermomixer (Eppendorf) with constant shaking (1,000 rpm) at room temperature for 1 h. For the negative control experiments, we also incubated six target bacterial cells (~10^7^ cells suspended in 1× PBS (100 μL)) with 3′-FAM dye-labelled random ssDNA (250 nM in final binding reaction, 5′-TGC CAC GGC GAA TGT CGG GGA GAC AGC AGC GAC TGC AGA CAT CAG ATC AGA GTA ATA CTA ACA TGC GAT AAG TCC C-FAM-3′). The incubated samples in 1× PBS (total 200 μL) were washed three times to remove unbound ssDNA with 1× PBS (1 mL), followed by centrifugation at 13,000 rpm at room temperature for 10 min. Samples were re-suspended in autoclaved water (50 μL). For the selectivity test, the binding of the individual ssDNA sequences to different cells (*P. putida, S. insulae, S. sonnei, E. hermannii, E. cloacae, K. alba, C. braakii, B. cereus*, and *S. aureus*) was performed using the same conditions. The fluorescence intensity of cells bound to 3′-FAM dye-labelled aptamers was measured using a micro-fluorospectrophotometer (NanoDrop-3300, Thermo SCIENTIFIC, Wilmington, DE, USA). The K_d_ (dissociation constant) was obtained by a non-linear regression analysis implemented in SigmaPlot 10.1 (Systat Software, Inc., San Jose, CA, USA).

To observe the binding of aptamers to target cell surfaces, the 3′-FAM dye-labelled aptamers or random ssDNA (500 nM in 1× PBS (100 μL)) were incubated with each bacterium (~2 × 10^7^ cells suspended in 1× PBS (100 μL)) for 1 h at room temperature with shaking (1,000 rpm). After incubation, the cells were washed three times with 1 mL of 1 × PBS (pH 7.0) to remove the unbound aptamers, followed by centrifugation (13,000 rpm) at room temperature for 10 min. Finally, the aptamer-cell complexes were re-suspended with 100 μL of autoclaved deionized water. The cells were then imaged using a fluorescence microscope (Olympus BX50, Olympus Corporation, Tokyo, Japan) 1 h after dropping the cell suspension on a slide glass.

## Additional Information

**How to cite this article**: Song, M. Y. *et al*. Broadly reactive aptamers targeting bacteria belonging to different genera using a sequential toggle cell-SELEX. *Sci. Rep.* **7**, 43641; doi: 10.1038/srep43641 (2017).

**Publisher's note:** Springer Nature remains neutral with regard to jurisdictional claims in published maps and institutional affiliations.

## Figures and Tables

**Table 1:** ssDNA sequences isolated using the STC-SELEX process.

ID	Sequence (5′–3′)	Size (bp)	Number of copies
STC-01	CATATCCGCGTCGCTGCGCTCAGACCCACCACTACGCACC	40	1
STC-02	CATATCCGTGTCGCTGCGCTCAGACCCACCACCACGCACC	40	1
STC-03	**CATATCCGCGTCGCTGCGCTCAGACCCACCACCACGCACC**	40	10
STC-04	ATATCCGCGTCGCTGCGCTCAGACCCACCACCACGCACC	39	1
STC-05	CATATCCGCGTCGCTGCGCTCAGACCCACCACCCGCCC	38	1
STC-06	GGGCGGGGGTGCTGGGGGAATGGAGTGCTGCGTGCTGCGG	40	6
STC-07	GGGCGGGGGGTGCTGGGGGAATGGAGTGCTGCGTGCTGCG	40	1
STC-08	GGGCGGGGGTGCTGGGAGAATGGAGTGCTGCGTGCTGCAG	40	1
STC-09	GGGCAGGTGTGCTGGGGGAATGGAGTGCTGCGTGCTGCGG	40	1
STC-10	AGACCGCAGGTGCACCGGGCGACGTCTCTGGGTGTGGTGA	40	1
STC-11	AGACCGCAGGTGCACTGGGCGACGTCTCTGGGTGTGGTGT	40	2
STC-12	**GGACCGCAGGTGCACTGGGCGACGTCTCTGGGTGTGGTGT**	40	8
STC-13	GGACGCGCGTTGGTGGTGGATGGTGTGTTACACGTGTTGT	40	5
STC-14	CGGGGTGGGACCAGTCTTGCGCGGGTGAC	29	1
STC-15	GGACTGGAGTCTAGACCGGGTAGCTGTGGT	30	2
